# Simulating diffraction photographs based on molecular dynamics trajectories of a protein crystal: a new option to examine structure-solving strategies in protein crystallography

**DOI:** 10.1107/S2052252522011198

**Published:** 2023-01-01

**Authors:** Ning Liu, Oleg Mikhailovskii, Nikolai R. Skrynnikov, Yi Xue

**Affiliations:** a School of Life Sciences, Tsinghua University, Beijing 100084, People’s Republic of China; b Laboratory of Biomolecular NMR, St Petersburg State University, St Petersburg, Russian Federation; c Department of Chemistry, Purdue University, West Lafayette, IN 47907, USA; d Beijing Advanced Innovation Center for Structural Biology, Tsinghua University, Beijing 100084, People’s Republic of China; e Tsinghua University–Peking University Joint Center for Life Sciences, Tsinghua University, Beijing 100084, People’s Republic of China; Chinese Academy of Sciences, China

**Keywords:** X-ray crystallography, molecular dynamics simulations, diffraction photographs, Huygens–Fresnel principle

## Abstract

A molecular dynamics (MD)-based pipeline has been designed and implemented to emulate the entire process of collecting diffraction photographs and calculating crystallographic structures of proteins from them. Using a structure of lysozyme solved in-house, a supercell comprising 125 (5 × 5 × 5) crystal unit cells containing a total of 1000 protein molecules and explicit interstitial solvent was constructed. For this system, two 300 ns MD trajectories at 298 and 250 K were recorded. A series of snapshots from these trajectories were then used to simulate a fully realistic set of diffraction photographs, which were further fed into the standard pipeline for structure determination. The resulting structures show very good agreement with the underlying MD model not only in terms of coordinates but also in terms of *B* factors; they are also consistent with the original experimental structure. The developed methodology should find a range of applications, such as optimizing refinement protocols to solve crystal structures and extracting dynamics information from diffraction data or diffuse scattering.

## Abstract 2

A molecular-dynamics-based pipeline has been designed and implemented to emulate the entire process of collecting diffraction photographs and calculating crystallographic structures of proteins from them. The developed methodology should find a range of applications, such as optimizing refinement protocols to solve crystal structures and extracting dynamics information from diffraction data or diffuse scattering.

## 1. Introduction

As an extremely powerful technique for structure determination of biomolecules, X-ray crystallography has led to nearly 90% of all 3D structures of proteins currently in the Protein Data Bank (PDB). Even under crystalline conditions, proteins are characterized by a considerable amount of dynamics on a broad range of timescales. This has profound implications not only for their diverse biological functions, but also for the interpretation of experimental data during the process of structure determination. Indeed, prior studies have long established the similarity between protein conformational dynamics in the solution and crystalline states (van Gunsteren & Berendsen, 1984 ▸; Agarwal *et al.*, 2008 ▸; Stocker *et al.*, 2000 ▸; Chevelkov *et al.*, 2010 ▸; Torchia, 2015 ▸). In particular, such a similarity is manifested by the fact that many enzymes maintain their activity in the crystal form (Bello & Nowoswiat, 1965 ▸; Makinen & Fink, 1977 ▸; Merli *et al.*, 1996 ▸). Hence, there has been considerable interest in molecular dynamics (MD) simulations of protein crystals, modeled by a single unit cell subject to periodic boundary conditions or, alternatively, by a block of multiple unit cells (referred to as a ‘supercell’). Such simulations offer a detailed picture of protein motions that is complementary to the experimental crystallographic results. Efforts to simulate protein dynamics in the crystalline state have been made since the early days of biomolecular MD simulations (van Gunsteren & Karplus, 1982 ▸; van Gunsteren *et al.*, 1983 ▸; van Gunsteren & Berendsen, 1984 ▸) and recent years have seen renewed interest in this area of research (Meinhold & Smith, 2005*a* ▸,*b* ▸; Janowski *et al.*, 2013 ▸; Cerutti & Case, 2019 ▸). Crystal MD simulations have been used to make connections to experimental observables, such as *B* factors (van Gunsteren *et al.*, 1983 ▸; Meinhold & Smith, 2005*a* ▸; Cerutti *et al.*, 2008 ▸, 2010 ▸; Kuzmanic *et al.*, 2014 ▸; Janowski *et al.*, 2016 ▸), NMR order parameters (Chevelkov *et al.*, 2010 ▸), X-ray free-electron laser (XFEL) patterns (Zook *et al.*, 2020 ▸) and diffuse scattering patterns (de Klijn *et al.*, 2019 ▸; Meisburger *et al.*, 2020 ▸).

Despite all of these accomplishments, to the best of our knowledge there have been no attempts to simulate the physical phenomenon of diffraction from first principles based on MD simulations of protein crystals. In previous studies, MD-extracted coordinates were used to calculate diffraction intensities only at Bragg peaks (van Gunsteren *et al.*, 1983 ▸; Kuriyan *et al.*, 1986 ▸; Gros *et al.*, 1990 ▸; Janowski *et al.*, 2013 ▸), rather than generating, pixel by pixel, a series of diffraction photographs as seen in the real world. At least in part, this situation can be attributed to the technical challenges of (i) recording an all-atom MD trajectory of a supercell of a sufficiently large size required to emulate crystal diffraction and (ii) generating a large number of high-resolution diffraction photographs from such a large-sized supercell simulation. These two tasks, which used to be intractable, are now within reach thanks to recent advances in computing hardware, *i.e.* the use of graphics processing unit (GPU) cards for general-purpose computations (Stone *et al.*, 2010 ▸).

Conventionally, the crystallographic diffraction pattern is calculated using direct summation methods or, otherwise, fast Fourier transform (FFT)-based methods (Janowski *et al.*, 2013 ▸; Gros *et al.*, 1990 ▸; Kuriyan *et al.*, 1986 ▸; van Gunsteren *et al.*, 1983 ▸; Kuzmanic *et al.*, 2014 ▸), producing a set of structure factors rather than diffraction photographs per se. However, the diffraction photographs can be generated in a straightforward manner based on the Huygens–Fresnel principle (Born *et al.*, 1999 ▸). Specifically, when an incident X-ray beam hits the crystal, each atom acts as a source of a secondary wavelet (Drenth & Mesters, 2007 ▸). The superposition of these wavelets on a distant receiver plane leads to the familiar diffraction pattern. Given an MD trajectory of a large crystal supercell, all atoms in each snapshot (including those from bulk solvent) can be treated as sources of wavelets, and the diffraction photograph can be easily generated pixel by pixel by proper averaging of wavelet contributions from all MD snapshots.

An alternative approach to simulate such photographs is to construct a 3D grid of electron density for each MD snapshot, average the resulting density maps over the entire trajectory and fast Fourier-transform (Cooley & Tukey, 1965 ▸) the averaged map into reciprocal space, from which the diffraction photographs at any desired rotation angle can quickly be obtained. The downside of this latter approach is the high demand for GPU memory, which sets a limit to the maximum size of the supercell and, in turn, compromises the precision of the simulated diffraction pattern. Apart from this, the two approaches are fundamentally equivalent.

In this work, we have simulated diffraction photographs based on MD simulations of a supercell comprised of 125 unit cells of tetragonal lysozyme. The calculations were conducted using the fundamental Huygens–Fresnel principle. Having generated an array of 180 diffraction images representing different rotation angles, we then used the standard suite of crystallography programs, *HKL*-2000 (Otwinowski & Minor, 1997 ▸)/*XDS* (Kabsch, 2010 ▸), *Coot* (Emsley *et al.*, 2010 ▸) and *Phenix* (Liebschner *et al.*, 2019 ▸), to process these images, extract structure factors and ultimately calculate protein coordinates using the experimental crystal structure as a molecular-replacement model. The procedure has been successful, resulting in reasonable structures and satisfactory quality metrics. Thus, we have demonstrated that a long state-of-the-art MD trajectory of a protein crystal can be used to emulate the entire process of crystallographic structure determination at an unparalleled level of realism, leading to an accurate structural model. We envisage that in the future this procedure could be used to interrogate the relationship between protein internal dynamics and crystallographic variables (for example *B* factors), to improve the computational tools used in the field of biomolecular crystallography and to validate and benchmark the different MD force fields used for biomolecular simulations.

## 2. Materials and methods

### 2.1. Crystallization, data collection and structure determination

Lysozyme from chicken egg white (Sigma, CAS 12650-88-3, catalogue No. L-6876) was dissolved in 0.1 *M* sodium acetate (pH 4.5, 4.6 or 4.7) to a final concentration of 20 mg ml^−1^. Crystallization was carried out by the hanging-drop vapor-diffusion method in a 24-well microplate sealed with vacuum grease. The reservoir solution consisted of 0.1 *M* sodium acetate pH 4.5, 4.6 or 4.7 and 0.9–1.1 *M* sodium chloride. 500 µl reservoir solution was added to the wells, while a drop consisting of 1 µl reservoir solution mixed with 1 µl protein solution at the matched pH was suspended on the inside of the lid. The 24-well microplate was then incubated at 16°C for crystal growth. Crystals appeared after one week.

A MicroMax-007 HF synchrotron source (Rigaku) was used for data collection at 100 K, and the *HKL*-2000 software was used to control data acquisition and process photographs. The distance between the center of the crystal and the detector was 45 mm. A total of 180 frames were collected with an exposure time of 20 s each, covering a rotation range of 180°. The coordinates for molecular replacement were from the PDB entry 193l (Vaney *et al.*, 1996 ▸). *Phenix* and *Coot* were subsequently used to refine the structural model based on the structure factors reported by *HKL*-2000. Finally, we obtained a crystal structure of lysozyme in space group *P*4_3_2_1_2 with unit-cell parameters *a* = *b* = 78.67, *c* = 36.93 Å (see Supplementary Table S1 for further details).

### 2.2. Supercell MD simulations

To compare the experimental and simulated diffraction photographs, we chose to simulate the same lysozyme crystal as investigated in our experimental study (see above). All MD simulations were performed using *AMBER* version 18 (Case *et al.*, 2018 ▸) with the ff14SB force field (Maier *et al.*, 2015 ▸) and the SPC/E water model (Berendsen *et al.*, 1987 ▸). Prior to simulation, *PROPKA* (Bas *et al.*, 2008 ▸) was used to predict the protonation states of Asp, Glu and His side chains. According to these predictions, residues Asp52 and Glu35 were converted to their respective protonated forms, Ash and Glh, respectively. In addition, all eight Cys residues were changed to Cyx, as they are known to form four disulfide bonds (Jaureguiàdell *et al.*, 1965 ▸).

To find the optimal dimensions of the simulation box, we first conducted a one-unit-cell crystal MD simulation at 100 K to determine the number of water molecules and ions leading to the correct cell dimensions (as found in our experimental study). This was accomplished by means of an iterative approach reported previously (Ma *et al.*, 2015 ▸). After this, we reran the one-unit-cell simulation at 250 K using the determined number of water molecules and ions. When this simulation reached equilibrium (after 100 ns), the resulting (slightly increased) dimensions of the simulation box (*a*′, *b*′ and *c*′) were taken to be the new unit-cell parameters at 250 K. Finally, we rebuilt the unit cell with the new dimensions (*a*′, *b*′ and *c*′) and the pre-determined number of water molecules and ions, and then energy-minimized the resulting system. The same procedure was used to construct the unit cell at 298 K.

The dimensions of the simulated unit cell increased by 0.7% when increasing the temperature from 100 to 250 K and by a further 0.6% when increasing the temperature from 250 to 290 K. The crystal lattices were constructed by the *UnitCell* command, which makes use of space-group symmetry operations, while water molecules and chlorine ions to neutralize the system were added by the *AddToBox* command. Both commands are part of the *AmberTools* suite (Case *et al.*, 2018 ▸). Following standard practices, we did not include other ions in the simulations (no ions are resolved in the experimental structure and, in fact, it remains unclear how ions are partitioned between the crystal and its mother liquor; also, the force-field parameters of the ions are not necessarily optimized to the same standard and harmonized with those for peptides and water).

The energy-minimized one-unit-cell box was then used to construct a 5 × 5 × 5 = 125 unit-cell supercell. Such 125-unit supercells with dimensions of 396.06 × 396.06 × 185.92 Å and 398.62 × 398.62 × 187.13 Å were used to record MD trajectories at 250 and 298 K, respectively. After energy-minimization and heating to the target temperature, the supercell simulations were equilibrated for 1 ns and then run for 300 ns under the NPT ensemble. The integration time step was set to 4 fs using hydrogen mass repartitioning (Hopkins *et al.*, 2015 ▸).

### 2.3. Diffraction photograph simulation

**Figure 1:**
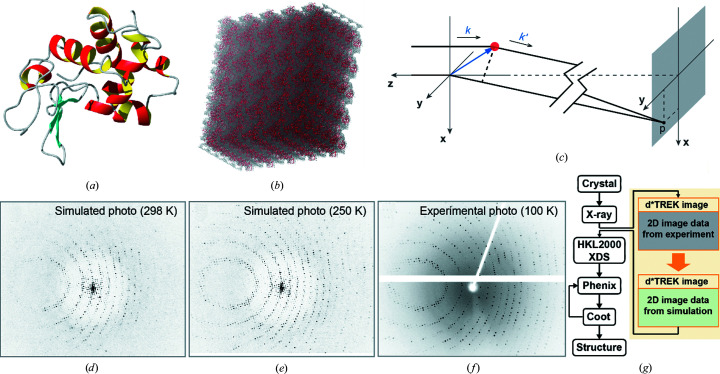
Generation of diffraction photographs from an MD trajectory of crystalline lysozyme. (*a*) The structure of lysozyme solved in-house (tetragonal lattice, 2.1 Å resolution). (*b*) A 5 × 5 × 5 supercell of crystalline lysozyme in the MD simulation, containing 1000 protein molecules, 391 250 water molecules and 10 000 Cl^−^ ions. (*c*) Diagram illustrating the calculation of an X-ray diffraction photograph based on the Huygens–Fresnel principle. The red dot represents the position of an atom in the crystal. **k** and **k**′ denote wavevectors before and after scattering, respectively. The point *p* represents a pixel on the receiver plane. (*d*) A simulated diffraction photograph from a 300 ns MD trajectory of the lysozyme crystal supercell at 298 K. (*e*) A simulated diffraction photograph from a 300 ns MD trajectory of the lysozyme crystal supercell at 250 K. (*f*) The experimental diffraction photograph taken at the same crystal rotation angle as the simulated photographs (*d*) and (*e*). (*g*) Flowchart of the structure back-calculation procedure using MD-based simulated diffraction photographs. In brief, the digitized diffraction images in the experimental data files were replaced with the simulated images, and these files were then used as input for the standard pipeline to solve the crystal structure, with our experimentally solved crystal structure as the molecular-replacement model.

The algorithm to simulate a diffraction photograph is illustrated in Fig. 1 ▸(*c*). The incident X-ray beam with wavelength λ and wavevector **k** is directed along the *z* axis. The position of a specific atom *j* in the crystal supercell is defined by **r**_*j*_. The electrons of this atom oscillate at the frequency of the incident wave and act as a source of secondary wavelets according to the Huygens–Fresnel principle. The direction of the light diffracted from atom *j* is indicated by the wavevector **k**′. The radiance received by a pixel *p* on the receiver plane is the sum of all rays diffracted by every atom in the supercell, including atoms of water molecules and ions. The H atoms were also taken into consideration, although their contribution is minimal. If we treat an optical path passing through the origin as a reference, then the phase difference for the light diffracted by atom *j* relative to the reference optical path is δ_*j*_ = 2π × **r**_*j*_ · (**k** − **k**′). Therefore, the diffracted light detected by pixel *p* becomes *A* *_j_* = *A*_0,*j*_exp(*i*δ*_j_*), where *A*_0,*j*_ is the amplitude of the diffracted wave. The resultant amplitude thus becomes

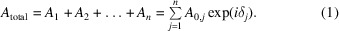




To compute the amplitude of the diffracted wave contributed by each atom, the scattering factor should be taken into consideration, leading to

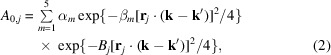

where α_*m*_ and β_*m*_ are atom-specific coefficients (Brown *et al.*, 2006 ▸) and *B_j_* is the *B* factor of atom *j* representative of the small-amplitude motion of the atom. In our approach, we assume that local atomic dynamics are adequately represented by a set of MD snapshots containing multiple protein and solvent molecules, thus obviating the need for *B* factors. Accordingly, we assigned a *B* factor of 0 to all atoms in the supercell. Note that the contributions of diffracted wavelets from all atoms in a given MD snapshot are summed in the form of complex numbers according to equation (1). On the other hand, to calculate the diffraction pattern over the trajectory, one needs to add the contributions from all snapshots by intensity (|*A*_total_|^2^) rather than in the form of complex numbers with phases. Indeed, it is known that intensities, but not phases, are registered in crystallographic experiments; it is also known that intensities increase with exposure time (imitated in our protocol by adding the intensities from sequential MD snapshots).

In the diffraction experiment on lysozyme crystallized in space group *P*4_3_2_1_2, we collected 180 diffraction photos; from these, we proceeded to solve the (very familiar) lysozyme structure. For consistency, we simulated the analogous 180 diffraction photos based on our supercell simulations. In order to place the supercell in a proper orientation (corresponding to the initial crystal orientation in our diffraction measurements) we developed a special algorithm that is described in the supporting information. The supercell (or, more precisely, the set of snapshots from the MD trajectory) was then rotated about the *x* axis [see Fig. 1 ▸(*c*)] and the diffraction images were generated with a step of Δ_Ω_ = 0.1°. These images were then combined, via |*A*_total_|^2^, in batches of ten to span the oscillation angle of 1° (the same as used experimentally). The procedure results in 180 simulated photos that cover a rotation range of 180°, imitating the experimental data set.

While our approach to simulation of diffraction photographs is highly realistic, it nevertheless involves certain simplifications. For example, we treat the diffraction wavelets as planar waves rather than spherical waves (far-field approximation). Of note, various instrumental factors affecting X-ray diffraction experiments have been discussed in considerable detail by Holton *et al.* (2014 ▸). These investigators concluded that the noise level in protein diffraction data is relatively low and is not a limiting factor with regard to the accuracy of crystallographic structures; moreover, the exact origin of the experimental noise remains unclear. Incorporation of the relevant instrumental effects into our simulation protocol, which should potentially lead to more realistic-looking diffraction photographs, is deferred to future studies.

The computational procedure described above is highly parallelizable and thus is suitable for GPU programming. When simulating a photograph with a size of 487 × 407 pixels, our CUDA code achieved an ∼700-fold acceleration compared with its counterpart written for a single CPU core. Such a speedup has proven to be crucial for simulating diffraction photographs from a supercell MD trajectory.

It is also appropriate to address certain potential risks associated with synthetic diffraction images here. Following some recent discoveries of fabricated crystallographic structures (Dauter & Baker, 2010 ▸; Else, 2022 ▸), the crystallographic community has increased its level of attention to data authenticity. In particular, calls have been made to include the original image data as a part of structure depositions (Hanson *et al.*, 2022 ▸). In this regard, we observe that the generation of synthetic diffraction images as described in this report requires a major commitment of computational resources, which provides effective insurance against potential misuse of our methodology.

### 2.4. Flowchart for structure back-calculation

To feed the simulated photographs into the standard pipeline for X-ray structure determination, we need to render them in a proper format. For this purpose, we took the experimental d*TREK files and replaced the original binary arrays, which encode the experimental diffraction images, with the corresponding simulated arrays. Within the simulated arrays, the image regions representing the screen spacer and the beamstop were copied from the experimental arrays. All other records in the d*TREK files were left unchanged. The obtained files were then used as input for the structure-solving protocol involving *XDS* (or *HKL*-2000), *Phenix* and *Coot* [see Fig. 1 ▸(*g*)]. Note that the simulated data arrays need to be rescaled to allow normal processing by *XDS*. To search for a suitable scaling factor, a semi-automated Python script has been implemented in-house. This script tests scaling constants between 0.1 and 10.0, applying them in a uniform manner to all simulated diffraction images. The scaled images are then loaded into *XDS* and the output file CORRECT.LP is consulted to identify the optimal scaling factor.

The structures calculated from the simulated diffraction data associated with the MD trajectories at 250 and 298 K, together with the experimentally solved lysozyme structure, are available as a part of the supporting information.

### 2.5. Data analysis

The calculation of the root-mean-square deviation (r.m.s.d.) between a supercell MD trajectory and a crystal structure used the definitions from a previous simulation study (Janowski *et al.*, 2016 ▸). To calculate the ‘best-fit r.m.s.d.’, we superpose all protein molecules in a given MD snapshot onto the experimental structure. To calculate the ‘lattice r.m.s.d.’, we align the entire supercell from a given MD snapshot with the initial 125-unit supercell built from the experimental crystal structure (achieved by aligning their respective centers of mass). The same scheme was used to calculate the atomic root-mean-square fluctuation (r.m.s.f.) for a set of MD frames, *i.e.* the ‘best-fit r.m.s.f.’ and ‘lattice r.m.s.f.’. Before calculating the lattice r.m.s.d. and r.m.s.f., one needs to correct for periodic jumps of protein molecules in the supercell simulation.

To validate the positions of ordered (crystallographic) waters in the experimental structure, as well as the back-calculated structure, we referred to hydrogen bonds formed between water molecules and proteins (Hubbard & Haider, 2010 ▸). In a hydrogen bond *D*—H⋯*A*, water can serve as either a donor or an acceptor of hydrogen. Here, we used two criteria, (i) the distance between atoms *D* and *A* should be less than 3.5 Å and (ii) the angle should be greater than 150°, to determine whether a hydrogen bond is formed. If a water molecule in two structures forms a hydrogen bond to the same protein atom (acting in the same capacity, for example that of a donor), we assume that these structures contain an identical ordered water molecule.

## 3. Results

As a starting point for this study, we solved the crystallo­graphic structure of tetragonal lysozyme. This provided a starting model for our crystal MD simulations and made it possible to draw a direct comparison between the experimental and simulated diffraction images. We grew crystals of chicken egg-white lysozyme at room temperature following the experimental protocol for the hallmark lysozyme structure PDB entry 193l (Vaney *et al.*, 1996 ▸) and solved the crystal structure using a standard pipeline consisting of *HKL*-2000, *Coot* and *Phenix*. The resulting structure [Fig. 1 ▸(*a*)] shows essentially the same crystallographic parameters as PDB entry 193l, with space group *P*4_3_2_1_2 and an r.m.s.d. to the target of 0.217 Å. This in-house structure was subsequently used as a building block to construct a supercell comprising 5 × 5 × 5 unit cells [Fig. 1 ▸(*b*)]. Next, we performed all-atom MD simulations of this supercell, which contained 1000 protein molecules, 391 250 water molecules and 10 000 chlorine ions.

One of the key questions is the choice of temperature(s) for MD simulations. Experimentally, the crystals were grown at room temperature before being rapidly cooled to 100 K for diffraction measurements. The cooling slows down the protein dynamics, but arguably preserves some of the conformational heterogeneity that is present at room temperature (for example, alternate rotameric states for some side chains). From a technical standpoint, the cooling occurs on a millisecond timescale, which is too long for an MD simulation. Furthermore, modeling crystalline dynamics at the low temperature of 100 K requires exceedingly long MD simulations. In this situation, we choose a compromise temperature of 250 K to record the supercell trajectory. On one hand, this is a subzero temperature, which to some degree approximates the experimental cryocooling conditions. On the other hand, this temperature is sufficiently high to sample protein dynamics and the concomitant conformational heterogeneity. For comparison, we also recorded a reference trajectory at room temperature (298 K).

For both temperatures, 250 and 298 K, we recorded 300 ns trajectories of the lysozyme supercell using *AMBER*18. The trajectories were recorded at a rate of ∼4.5 ns per day on RTX 2080ti GPU cards. During the simulations the protein coordinates stabilized in a reasonably short amount of time, as shown by the best-fit r.m.s.d. traces [Supplementary Figs. S1(*a*) and S1(*b*)]. The lattice as a whole also remained fairly stable at 250 K, although at 298 K it was still adjusting towards the end of the trajectory [Supplementary Figs. S1(*c*) and S1(*d*)]. On a related note, we previously found that MD models of protein crystals tend to have lower stability at room temperature (Ma *et al.*, 2015 ▸; Kurauskas *et al.*, 2017 ▸).

Next, we set out to simulate the diffraction photographs from the MD trajectories. After discarding the initial 100 ns, we extracted ten (uniformly spaced) frames from each trajectory. Given that every frame contains 1000 protein molecules and a matching amount of interstitial water, this set of frames provides an excellent sample of crystal dynamics. Each frame was placed in exactly the same orientations as the actual crystal during the course of diffraction measurements (the orientations were determined by analyzing the *HKL*-2000 output from the processing of the experimental diffraction photographs; see the supporting information). After this, we combined the contributions from the secondary wavelets originating from all atoms in the supercell, as described in Section 2.3. In this manner, we obtained the intensity at the position of a certain specific pixel in the diffraction photograph [Fig. 1 ▸(*c*)]. Adding the pixel intensities from the individual frames ultimately leads to a simulated photograph with dimensions of 487 × 407 pixels. It is worth noting that equivalent results can in principle be obtained using a powerful FFT technique. However, as already mentioned, this would require more memory than is currently available on a standard GPU workstation.

For each of the two trajectories, we thus produced a series of 180 simulated photographs corresponding to the crystal being rotated around the *x* axis with a step of 1°. The simulated photographs for a rotation angle of 90° are shown in Figs. 1 ▸(*d*) and 1 ▸(*e*), with the corresponding experimental photograph shown in Fig. 1 ▸(*f*). Not surprisingly, more Bragg peaks are visible in the high-resolution shells of the photograph from the 250 K trajectory than the photograph from the 298 K trajectory. This can be readily explained by the elevated level of dynamics in the high-temperature simulation (Supplementary Fig. S1). Despite the overall similarity of the simulated and experimental diffraction patterns, the intensities of the individual reflections show only a modest level of correlation. This is due to the appreciable structural differences between the MD model of the crystal and the original crystallographic coordinates (reflected in Supplementary Fig. S1; see below for additional details).

**Figure 2:**
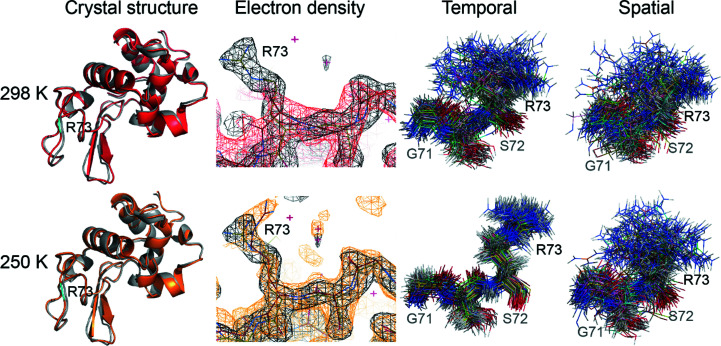
The side-chain dynamics of residue Arg73 in time representation and ensemble representation. First column: comparison of the experimental crystal structure (gray) and the recovered structures solved using the simulated diffraction data obtained from the MD trajectories at 298 K (red) and 250 K (orange). Residue 73 in the recovered structures is highlighted in cyan. Second column: electron density of residues 71–73 in the recovered structures (red for 298 K, orange for 250 K) and in our experimental structure (gray). Third column: the temporal ‘bundles’ containing 125 lysozyme molecules extracted from the uniformly spaced MD frames at 298 and 250 K (the frames sample the final two-thirds of the trajectory from 100 to 300 ns). Fourth column: the ensemble (spatial) ‘bundles’ containing 125 lysozyme molecules extracted from the last MD frame at 298 and 250 K. Note that for the trajectories that have not fully converged the thus-defined spatial bundles should be broader than the corresponding temporal bundles.

Using the set of simulated diffraction photographs, we were able to carry out the standard procedure for solving the crystal structure. For this purpose, we modified the experimental d*TREK files, grafting in the simulated images [in the form of binary arrays; see Fig. 1 ▸(*g*)]. Either *HKL*-2000 or *XDS* can be used to process such files, and these two programs indeed produced similar results in our tests; in the end, we opted for *XDS* because of its well developed control options. Using a pipeline involving *XDS*, *Phenix* and *Coot*, we calculated the structure of lysozyme to resolutions of 1.89 and 2.15 Å from the trajectories at 250 and 298 K, respectively (see Fig. 2 ▸, left column; see also Table 1 ▸ and Supplementary Table S1 for detailed information). The resulting *R*_work_ and *R*_free_ values are 0.170 and 0.211, respectively, for the 250 K structure and 0.189 and 0.225, respectively, for the 298 K structure. The respective *MolProbity* (Williams *et al.*, 2018 ▸) scores are 1.34 and 1.46 (both corresponding to the 98th percentile within the relevant resolution range).

Remarkably, the structures obtained from the MD-based simulated diffraction data are in excellent agreement with the underlying MD trajectories. Considering C^α^ atoms, the 250 K structure is within 0.14 Å of the average MD coordinates, while the 298 K structure is within 0.26 Å of the average MD coordinates. The ability to correctly reproduce the coordinates of the protein backbone signifies the success of the described ‘imitation crystallography’ scheme.

The comparison is less straightforward for side chains because they tend to sample different rotameric states (in which case average MD coordinates are not necessarily very meaningful). Nevertheless, the agreement remains reasonably good, as shown by the all-atom r.m.s.d. between the recovered structures and the average MD coordinates: 0.62 Å for the 250 K structure and 0.72 Å for the 298 K structure.

The two back-calculated structures also overlay relatively well with the experimental structure, with a C^α^ r.m.s.d. of 0.5 Å and a heavy-atom r.m.s.d. of 1.1 Å for the 250 K structure (0.6 and 1.2 Å for the 298 K structure). This is consistent with the deviations between the MD-averaged coordinates and the experimental structure: 0.5 and 0.9 Å for the 250 K trajectory (0.6 and 1.0 Å for the 298 K trajectory). How should we interpret these results? On one hand, a backbone r.m.s.d. of the order of 0.5 Å indicate that the two structures are near-identical. On the other hand, for a pair of medium-resolution structures solved in the same crystal form the backbone r.m.s.d. is typically lower, in the range of 0.2–0.3 Å (Eyal *et al.*, 2005 ▸). As already mentioned, this result reflects the systematic divergence between the actual crystal structure and its MD representation. It should be noted that the phenomenon of ‘structural drift’ in protein MD simulations has been well documented. In particular, Shaw and coworkers addressed this issue in a rather general manner using ultralong MD simulations (Raval *et al.*, 2012 ▸). Furthermore, Case and coworkers, as well as Li and coworkers, investigated this problem specifically with regard to MD models of protein crystals (Li *et al.*, 2014 ▸; Cerutti *et al.*, 2010 ▸). While the simulated crystal lattice remains visually intact, small distortions build up as a function of time: individual protein molecules become displaced from their positions (defined by crystal symmetry), change their orientation relative to the crystallographic axes and also develop systematic differences in terms of their internal structure. It is this behavior that is responsible for (i) the substantial r.m.s.d. between the MD-based recovered structure and the original crystallographic structure, as well as (ii) the relatively poor correlation between the respective structure factors (Supplementary Fig. S2).

One statistic that deserves a separate discussion is *R*_merge_, which is widely used as a measure of precision for experimental crystallographic data (Holton *et al.*, 2014 ▸; Wang *et al.*, 2017 ▸). In our recovered structures, this parameter is only borderline acceptable: 0.105 and 0.136 for the structures at 250 and 298 K, respectively (Supplementary Table S1). What is the source of this rather poor statistic? We have found that in our protocol the value of *R*_merge_ is sensitive to sampling of the oscillation angle Δ_Ω_ that is used to simulate the diffraction photographs (see Section 2.3).

To illustrate this effect, we built an ideal 125-unit supercell (fully obeying the crystal symmetry), solvated it with randomly positioned waters and generated three sets of diffraction photographs from it using different Δ_Ω_ steps. The simulated data were subsequently processed by *XDS* in order to determine *R*_merge_. It turns out that the coarse step of 1° leads to an extremely poor statistic, *R*_merge_ = 0.260, the default step of 0.1° produces a significant improvement, *R*_merge_ = 0.172, and the fine step of 0.01° further improves the outcome to *R*_merge_ = 0.085, bringing it to the level that is generally considered to be acceptable (Wlodawer *et al.*, 2013 ▸).

A similar trend is observed with our MD frames, where using a coarse step of 1° leads to a sharp deterioration of *R*_merge_, as well as substantial increases in *R*_work_ and *R*_free_ (not shown). The results are improved by switching to a 0.1° step, but do not change any further when employing the fine step of 0.01°. We assume that crystalline dynamics, as transpires in the MD simulation, becomes a limiting factor here. On one hand, it appears that the presence of dynamics mitigates the effect of coarse Δ_Ω_ sampling. On the other hand, the structural drift that occurs during the MD simulation and degrades the symmetry of the simulated crystal lattice is likely to be responsible for the elevated values of *R*_merge_ at 250 K and especially at 298 K.

Another interesting aspect of structure calculations concerns the recovery of ordered (crystallographic) waters. In previous studies, partial success has been achieved in recovering crystallographic waters from MD simulations of protein crystals (Wall *et al.*, 2019 ▸; Caldararu *et al.*, 2020 ▸). Here, we identified 121 ordered water molecules in the electron-density map of the recovered structure from the 250 K trajectory. Among them, 56 water molecules are hydrogen-bonded to the protein in the same way as in the experimental structure. For comparison, 42 ordered water molecules have been identified in the recovered structure derived from the 298 K trajectory, with 33 of them matching those in the experimental structure. The difference apparently stems from the enhanced dynamics caused by the higher temperature. The same effect is also evident in the electron density of side chains. For instance, a mobile side chain of residue Arg73 does not appear in the density map generated from the 298 K trajectory even after decreasing the contour level, whereas the same side chain is clearly visible in the density map at 250 K (Fig. 2 ▸, second column).

It is interesting to compare the time representation and ensemble representation of protein conformational dynamics in our crystal simulation. For the purpose of visualization, we generated structure bundles from the two MD trajectories either using 125 protein molecules from the last frame of the trajectory (one protein per unit cell) or using 125 copies of the same protein molecule extracted from the uniformly spaced MD frames (one copy per frame). The bundles are illustrated in Fig. 2 ▸ (third and fourth columns). For the fully equilibrated and converged trajectory we expect that the temporal bundle and ensemble bundle should have a similar appearance (as follows from the ergodicity principle). In Fig. 2 ▸ this is not quite the case, which suggests a lack of convergence with regard to the particular side chain illustrated in this plot. For the actual crystal, rapid cooling may lead to a kinetically trapped species (for example with regard to side-chain conformations); in this sense it is possible that during the diffraction measurements the crystal cannot be regarded as a fully equilibrated system.

Now that we know that the crystal structure can successfully be recovered using our simulated diffraction photographs, we ask ourselves whether the *B* factors (which are the main measure of crystalline dynamics) can be reproduced similarly well. There is a well known relationship between the *B* factors and the amplitude of atomic fluctuations, 

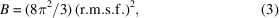

where r.m.s.f. stands for the atomic root-mean-square fluctuation (Willis & Pryor, 1975 ▸). Note, however, that strictly speaking this relationship only applies when the atomic fluctuations are describable by a Gaussian function, *i.e.* for small-amplitude motions controlled by a harmonic potential (Na *et al.*, 2021 ▸). Therefore, first we set out to test this relationship by comparing the *B* factors from our recovered (MD-based) structures, *B*_sim_, with those directly calculated from the MD trajectories by means of equation (3), *B*_r.m.s.f._. For the purpose of this comparison we use the lattice r.m.s.f., which accounts for all forms of crystal dynamics, including internal protein dynamics as well as small movements of protein molecules within the confines of a crystal lattice. The only motional mode that is factored out is the mass transfer across the boundaries of the simulation cell (see Section 2), which is a feature of the MD protocol and has no relevance in the real world.

**Figure 3:**
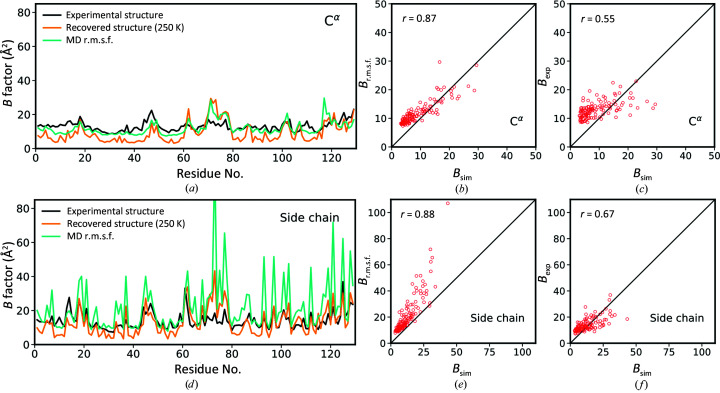
Crystalline dynamics as reflected in the *B* factors using the simulations at 250 K. (*a*) *B* factors of C^α^ atoms as found in our experimental lysozyme structure (*B*_exp_, black profile) and recovered lysozyme structure (*B*_sim_, orange profile), as well as calculated from the MD trajectories using equation (3) (*B*_r.m.s.f._, cyan profile). (*b*, *c*) Correlation plots *B*_r.m.s.f._ versus *B*_sim_ and *B*_exp_ versus *B*_sim_ for C^α^ atoms. (*d*) Mean side-chain *B* factors *B*_exp_, *B*_sim_ and *B*_r.m.s.f._ plotted as a function of residue number. (*e*, *f*) Correlation plots *B*_r.m.s.f._ versus *B*_sim_ and *B*_exp_ versus *B*_sim_ for mean side-chain *B* factors. The calculations of *B*_r.m.s.f._ used the same set of ten MD frames that was used to simulate diffraction photographs. The analogous results from 298 K simulations are shown in Supplementary Fig. S3.

The results of the comparison for the simulations at 250 K are illustrated in Fig. 3 ▸. In particular, Fig. 3 ▸(*a*) reports the *B* factors of C^α^ atoms plotted as a function of residue number. The *B*_sim_ profile in this graph (orange curve) closely follows the *B*_r.m.s.f._ profile (cyan curve), albeit with a small offset. The correlation between *B*_sim_ and *B*_r.m.s.f._ is very good, with Pearson correlation coefficient *r* = 0.87 [see Fig. 3 ▸(*b*)]. It is worth noting that the same kind of correlation between *B*_sim_ and *B*_r.m.s.f._ at 298 K is also very good, with *r* = 0.84 (see Supplementary Fig. S3).

In addition, Fig. 3 ▸(*d*) reports mean side-chain *B* factors, *i.e.* *B* factors averaged over all heavy atoms in a given side chain. Similar to the backbone results above, the *B*_sim_ profile in this plot matches closely the *B*_r.m.s.f._ profile, yielding a correlation coefficient *r* = 0.88. However, the latter profile features a number of spikes, where *B*_r.m.s.f._ values are twofold to threefold higher than the corresponding *B*_sim_ values. This behavior is characteristic of highly mobile side chains that undergo rotameric jumps (among them is the side chain of Arg73, which has already been discussed). In such cases the standard relationship expressed by equation (3) becomes problematic. One can argue that the empirical correlation between *B*_sim_ and *B*_r.m.s.f._, shown in Fig. 3 ▸(*e*), offers a generalization of this relationship, which is applicable not only for conformationally constrained side chains but also for those that jump between different rotameric states. A detailed investigation of how side-chain dynamics is reflected in crystallographic *B* factors, including those side chains that are modeled with alternate conformations, is deferred to future studies.

Next, we compare *B*_sim_ with the *B* factors from the experimentally solved lysozyme structure, *B*_exp_ [black profiles in Figs. 3 ▸(*a*) and 3 ▸(*d*)]. Since we already know that MD coordinates deviate appreciably from the experimental crystallographic coordinates (see above), we expect to find similar deviations between the respective motional amplitudes. Indeed, the correlation between *B*_sim_ and *B*_exp_ is only mediocre, with *r* = 0.55 and 0.67 for the backbone and side chains, respectively [see Figs. 3 ▸(*c*) and 3 ▸(*f*)]. One should bear in mind, however, that the experimental diffraction data were collected at a temperature of 100 K, whereas the simulations were conducted at 250 K; while it has only a limited impact on atomic coordinates, it may have a greater influence on motional amplitudes.

To summarize our observations in this area, crystallographic *B* factors indeed contain rich and accurate information about crystalline dynamics [Figs. 3 ▸(*b*) and 3 ▸(*e*)]. However, MD simulations of protein crystals show only so-so agreement with the actual experimental results [Figs. 3 ▸(*c*) and 3 ▸(*f*)]. In the future, the situation may be improved through the development of better force fields suited for protein crystal simulations (particularly at low temperatures) and the arrival of more powerful computers.

## 4. Concluding remarks

Several programs to simulate diffraction images have been reported in the past (Kolatkar *et al.*, 1994 ▸; Diederichs, 2009 ▸). It is particularly instructive to draw a comparison to the most recent such program, *MLFSOM* (Holton *et al.*, 2014 ▸). This program uses structure factors calculated from protein coordinates to obtain diffraction photographs; the disordered solvent is modeled using a flat mask-based solvent model. The shape of diffraction spots is calculated by taking into consideration a number of experimental factors contributing to the rocking curve (beam divergence, spectral dispersion, mosaic spread and point-spread function of the detector). To simulate the background, Holton and coworkers included multiple contributions such as scattering from air, diffuse scattering from disorder in the crystal lattice *etc.* Generally, the authors made a strong effort to incorporate various experimental effects into their calculations and to discuss various potential sources of uncertainty in the obtainable data.

Similar to our work, *MLFSOM* was tested on a tetragonal crystal of hen egg-white lysozyme (Holton *et al.*, 2014 ▸). The crystallographic structure of lysozyme solved by the authors was used to simulate a set of diffraction photographs, which were subsequently employed to recover the coordinates of the protein. Of particular interest, the authors found that experimental errors (*i.e.* the noise in the structure-factor data) as well as potential shortcomings of the structure-calculation protocol (*i.e.* the so-called phase bias) cause only fairly minor deviation between the ‘observed’ (simulated) and back-calculated structure factors. The corresponding *R*_work_ and *R*_free_ values were as low as 0.038 and 0.055, respectively. This led the authors to conclude that ‘the reason for high *R* factors in macromolecular crystallography is neither experimental error nor phase bias, but rather an underlying inadequacy in the models used to explain our observations’. Specifically, this inadequacy is due to ‘the present inability to accurately represent the entire macromolecule with both its flexibility and its protein–solvent interface’. Holton and coworkers predict that in the future this deficiency may be remedied by synergies between crystallography, simulations and small-angle X-ray scattering, allowing one to exploit the ‘substantial hidden and untapped potential’ contained in macromolecular diffraction data, *i.e.* to probe the dynamic dimension of protein crystals.

We believe that our study constitutes a step towards this goal. Indeed, instead of a single static structure we have used a uniquely large MD model of a protein crystal (1000 protein molecules, 300 ns simulation), offering a highly realistic representation of protein conformational diversity (‘flexibility’). Not only have we simulated the crystal with explicit solvent, but we have also used the explicit solvent coordinates to directly evaluate the solvent contribution to each individual pixel in the diffraction photograph. In this manner, we avoid the division of solvent into ordered water and bulk water and further avoid modeling the latter via one of the mask-based continuum models. This clearly constitutes a more rigorous approach to the modeling of the all-important ‘protein–solvent interface’.

Our recovered structures are in very good agreement with the underlying MD trajectories that have been used to simulate the diffraction data, with a C^α^ r.m.s.d. of the order of 0.1–0.3 Å. The agreement with the experimental structure is not quite so good at 0.5–0.6 Å. Similar observations can be made about the recovered *B* factors, which correlate very well with the underlying MD data, *r* ≃ 0.9, but not so well with the experiment, *r* ≃ 0.6. This behavior is due to the appreciable divergence between the MD model of the crystal and the actual crystal.

Such systematic divergence effects (Raval *et al.*, 2012 ▸) should become smaller with future improvements in force fields, leading to increasingly realistic diffraction images. At the same time, rapid progress in the area of computer hardware should enable a number of computational enhancements, including the use of FFT-based methods, as well as larger MD simulation cells and longer trajectories. All of this should also facilitate the modeling of protein crystals at lower temperatures, approximating the actual experimental conditions.

A number of potential applications can be envisioned for the new methodology described in this report. For instance, one can use a supercell MD simulation as the refinement target when imitating the entire process of structure determination. This may help to improve the existing refinement strategies, in particular with respect to the extraction of dynamics parameters such as *B* factors. On a related note, our simulation scheme can be used to elucidate the relationship between the experimentally determined *B* factors and the underlying protein motions, including side-chain rotameric jumps and conformational rearrangements in the loop regions. Broadly speaking, the proposed method makes it possible to assess the ‘true accuracy’ of crystallographic models in relation to various parameters such as crystallographic resolution, *R* factors *etc.* Other intriguing applications include the simulation of diffraction photographs in the context of XFEL crystallography, which makes use of tiny crystals and is therefore well suited to the methodology developed in this work, and diffuse scattering, which manifests itself in the diffraction photographs as a low-intensity background pattern believed to be linked to various dynamic modes characteristic of the crystal lattice (Meisburger & Ando, 2017 ▸; Van Benschoten *et al.*, 2015 ▸).

## 5. Related literature

The following reference is cited in the supporting information for this article: Ewald (1969 ▸).

## Supplementary Material

Supporting Methods, Supplementary Figures and Supplementary Table. DOI: 10.1107/S2052252522011198/lz5062sup1.pdf

The experimental lysozyme PDB structure and the two recovered PDB structures from MD. DOI: 10.1107/S2052252522011198/lz5062sup2.zip

## Figures and Tables

**Table 1:** Structure information

Structure	Temperature (K)	R.m.s.d.† (Å)	R.m.s.d.‡ (Å)	Resolution§ (Å)	*a*, *b*, *c* ¶ (Å)	*R*_work_ ††	*R*_free_ ††	Water‡‡
Experiment	100	0/0	0.516/0.948§§	2.10	78.666, 78.666, 36.928	0.173	0.223	138/138
			0.587/1.040¶¶					
MD-based	250	0.507/1.134	0.135/0.622	1.89	79.216, 79.216, 37.189	0.170	0.211	56/121
MD-based	298	0.562/1.208	0.264/0.724	2.15	79.723, 79.723, 37.413	0.189	0.225	33/42

† The best-fit r.m.s.d. is calculated for C^α^ atoms (left number) or heavy atoms (right number) relative to the crystal structure.

‡ The best-fit r.m.s.d. is calculated for C^α^ atoms (left number) or heavy atoms (right number) relative to the average MD coordinates.

§ Resolution is from the *XDS* output file CORRECT.LP.

¶ Unit-cell dimensions (α, β and γ are 90° for space group *P*4_3_2_1_2).

†† Crystallographic *R* factors as reported by *Phenix* following 20–22 cycles of standard refinement.

‡‡ Total number of ordered water molecules in the structure (right number), including those that also appear in the experimental crystal structure (left number).

§§ To average MD coordinates at 250 K.

¶¶ To average MD coordinates at 298 K.
