# Supplementary material for: Simulating diffraction photographs based on molecular dynamics trajectories of a protein crystal: a new option to examine structure-solving strategies in protein crystallography
Source: IUCrJ. 2023 Jan 1;10(Pt 1):16–26. doi: 10.1107/S2052252522011198 (PMC9812212; doi:10.1107/S2052252522011198)
Supplement: Supplementary file 1 [file m-10-00016-sup1.pdf]

IUCrJ

Volume 10 (2023)

Supporting information for article:

Simulating diffraction photographs based on molecular-dynamics trajectories of a protein crystal: a new option to examine structure-solving strategies in protein crystallography

Ning Liu, Oleg Mikhailovskii, Nikolai R. Skrynnikov and Yi Xue

Supporting Methods

Fitting rotation angles from the diffraction photographs

The objective here is to extract the orientation of the crystal from the experimental diffraction photograph – and then place the model crystal in exactly the same orientation (i.e. reorient all MD frames accordingly). First, the Miller indices and their positions in the photo were read from ".x" file, a type of auxiliary file generated by HKL-2000 when processing the experimental photos. Second, the center of Ewald sphere was determined by satisfying the simple geometry relationship illustrated in the diagram below (Ewald, 1969). Assuming that n triplets of Miller indices can be identified from a photo, the following equations should be fulfilled:

$$\left|\overline{s_i} - \overline{s_c}\right|^2 = 1/\lambda^2,\ i = 1, 2, 3, \cdots, n. \qquad \text{(S1)}$$

Here λ is the wavelength, $\overrightarrow{s_c}$ represents the location of the center of Ewald sphere, and $\overrightarrow{s_i}$ is the reciprocal vector corresponding to the ith triplet of Miller indices: $\overrightarrow{s_i} = h_i \cdot \overrightarrow{a^*} + k_i \cdot \overrightarrow{b^*} + l_i \cdot \overrightarrow{c^*}$, where $\overrightarrow{a^*}, \overrightarrow{b^*}, \overrightarrow{c^*}$ are three reciprocal basis vectors.

The above set of equations can be readily linearized with respect to the components of $\overrightarrow{s_c}$. This has been accomplished by subtracting the first equation from every other equation in the set. The resulting system of linear equations can be easily solved to obtain $\overrightarrow{s_c}$. Next, we determine the rotation matrix that rotates $\overrightarrow{s_c}$ to align it with the reference vector associated with the incident beam, (0, 0, $1/\lambda$) (see Fig. 1). From this matrix we extract the three axis-rotation angles, φ_x, φ_y and φ_z, specifying the orientation of the crystal. However, the obtained angle φ_z is arbitrary, as the final rotation about the z-axis does not change the orientation of the rotated vector (see Fig. 1). Therefore, an additional step is needed to determine the orientation of the crystal. To this end, we adjust φ_z seeking to match the calculated locations of Bragg peaks with those in the experimental photograph. This is achieved through a non-linear fitting algorithm implemented in a Python script that invokes the function *scipy.optimize.leastsq*.

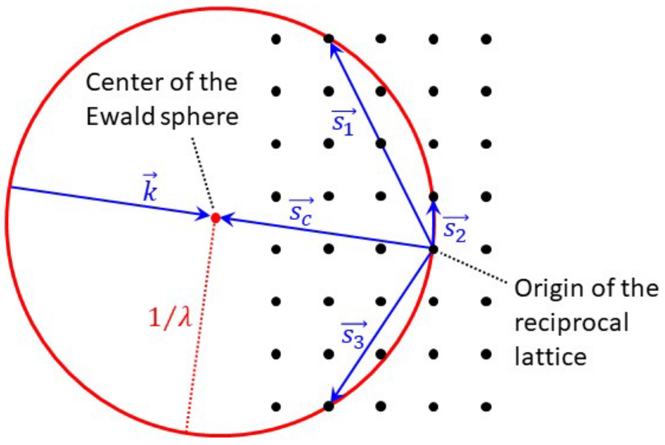

Supporting Figures

Figure S1. r.m.s.d. traces of the two MD trajectories relative to the experimental crystal structure: (a) best-fit r.m.s.d. for C^{α} atoms; (b) best-fit r.m.s.d. for all heavy atoms; (c) lattice r.m.s.d. for C^{α} atoms; (d) lattice r.m.s.d. for all heavy atoms. The best-fit r.m.s.d. was obtained by superposing each of the 1,000 protein copies in the MD frame onto the experimental structure and then averaging the results. The lattice r.m.s.d. was obtained by aligning (via the center of mass) the entire arrangement of 1,000 protein molecules from the MD frame with and the ideally arranged 5 × 5 × 5 crystallographic supercell.

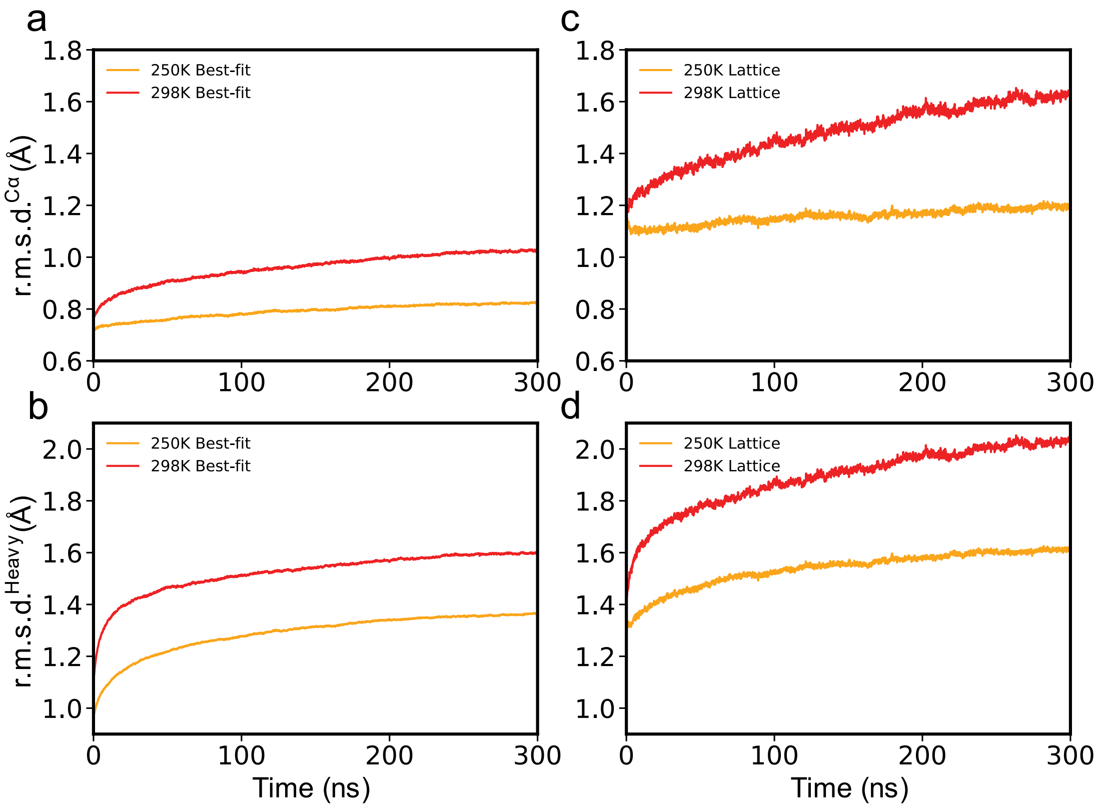

Figure S2. The correlation plot for structure factors from the experimental crystallographic structure of lysozyme (SF_{exptl}) and the corresponding recovered structures (SF_{sim}). All structure factors have been calculated using Phenix.fmodel command in the Phenix package. R-factors have been computed using the standard definition, $R = \frac{\sum|SF_{sim} - SF_{exptl}|}{\sum SF_{sim}}$.

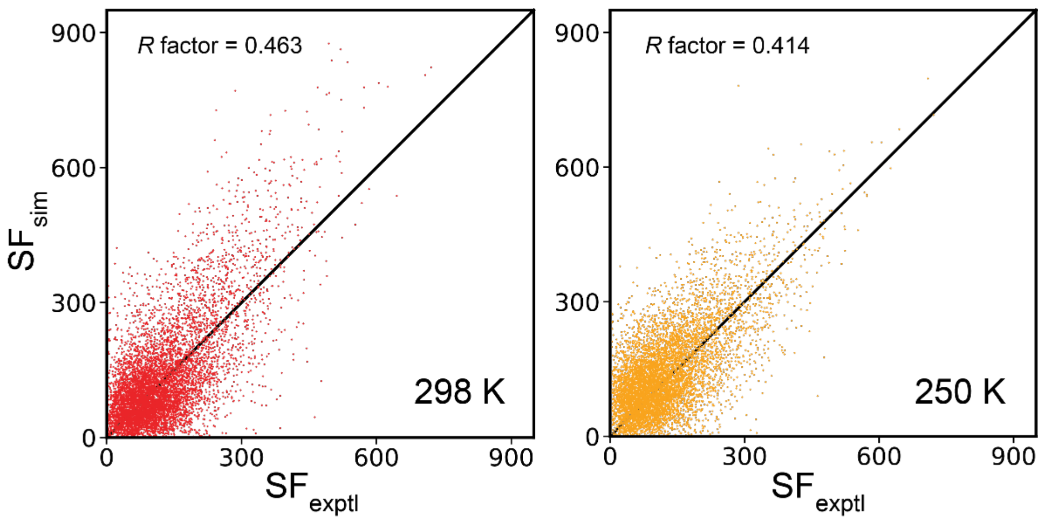

Figure S3. Crystalline dynamics as reflected in the B-factors using the simulations at 298 K. Plotting conventions are the same as in Fig. 3 in the main text.

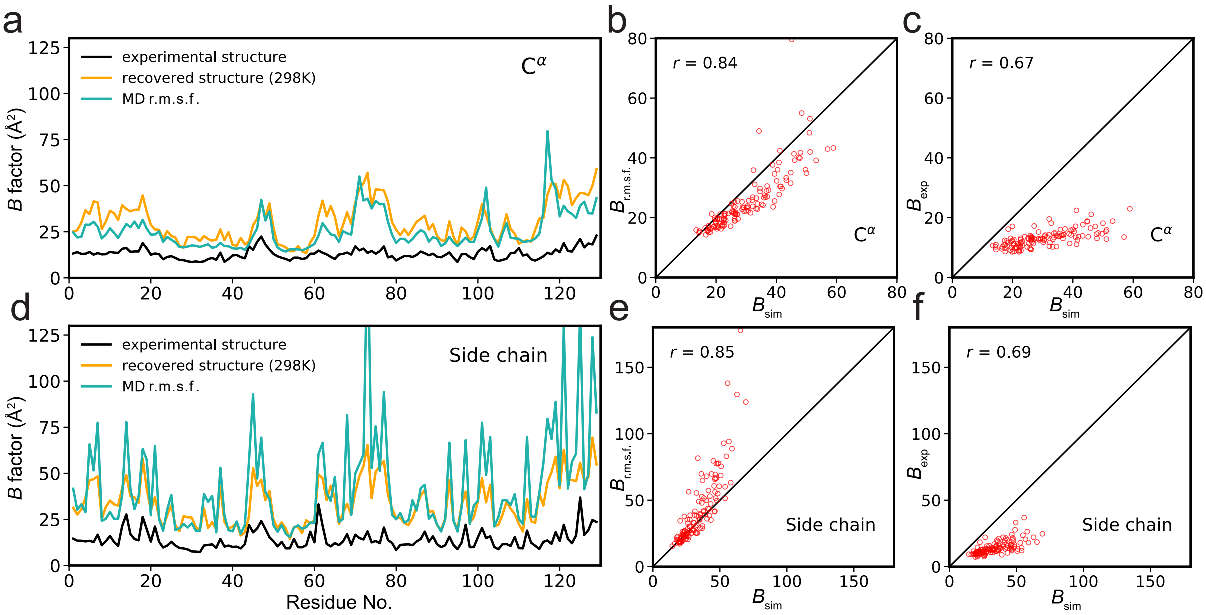

Supporting Tables

Table S1. Data collection and refinement of lysozyme experimental structure and recovered structures.

	Experiment (100 K)	MD (250 K)	MD (298 K)
**Data collection**			
Wavelength [Å]	1.5418	1.5418	1.5418
Space group	P $4_3 2_1 2$	P $4_3 2_1 2$	P $4_3 2_1 2$
**Cell dimensions**			
a, b, c [Å]	78.67, 78.67, 36.93	79.22, 79.22, 37.19	79.72, 79.72, 37.41
α, β, γ [°]	90, 90, 90	90, 90, 90	90, 90, 90
Resolution [Å]	50-2.10 (2.14-2.10)	56.01-1.89 (2.00-1.89)	56.37-2.15 (2.28-2.15)
R_{merge}	0.070 (0.157)	0.105 (0.176)	0.136 (0.441)
$I/\sigma I$	26.88 (2.81)	16.55 (5.57)	12.94 (4.91)
Completeness [%]	98.0 (84.4)	94.4 (70.2)	99.8 (99.3)
Redundancy [%]	12.0 (10.2)	11.0 (20.0)	14.2 (46.2)
**Refinement**			
Resolution [Å]	39.333-2.100 (2.175-2.10)	35.426-1.890 (1.990-1.890)	33.869-2.148 (2.314-2.148)
No. of reflections	7175 (711)	9381 (939)	6953 (696)
R_{work}	0.173 (0.190)	0.170 (0.187)	0.189 (0.216)
R_{free}	0.223 (0.244)	0.211 (0.241)	0.225 (0.268)
**No. of non-H atoms**			
Protein	1009[1]	1001	1001
water	138	121	42
**B-factors**			
Protein	14.02	12.13	32.90
water	21.56	24.95	35.27
**r.m.s. deviations**			
Bond lengths [Å]	0.06	0.008	0.009
Bond angles [°]	0.76	0.870	0.954
MolProbity score	0.77 (100th percentile)	1. 34 (98th percentile)	1.46 (98th percentile)

1. The quality of experimental electron density allowed us to resolve three side chains with alternate conformations, resulting in slightly larger number of heavy atoms than in the recovered structures.

References

Ewald, P. (1969). *Acta Crystallographica Section A* **25**, 103-108.
